# Dermatology Scheduling Triage of Transplant Patients and Transplant Candidates to Improve Early Diagnosis and Prevention of Skin Cancer: International Immunosuppression and Transplant Skin Cancer Collaborative Expert Consensus Recommendations

**DOI:** 10.3389/ti.2025.14711

**Published:** 2025-09-11

**Authors:** Kelsey E. Hirotsu, Lauren Crowe, Basia Michalski-McNeely, Sarah T. Arron, Kristin Bibee, Matthew J. Bottomley, David R. Carr, Joi B. Carter, Sean R. Christensen, Christina Chung, Anokhi Jambusaria, Kimberly M. Ken, Manisha J. Loss, Gyorgy Paragh, Elsemieke I. Plasmeijer, Charlotte Proby, Melissa Pugliano-Mauro, Kathryn T. Shahwan, Melodi Javid Whitley, Bryan T. Carroll

**Affiliations:** ^1^ Department of Dermatology, Stanford University School of Medicine, Redwood City, CA, United States; ^2^ Dermatology Associates of Tallahassee, Tallahassee, FL, United States; ^3^ Division of Dermatology, Washington University in St. Louis, St. Louis, MO, United States; ^4^ Palo Alto Foundation Medical Group, Palo Alto, CA, United States; ^5^ Department of Dermatology, University of Virginia, Charlottesville, VA, United States; ^6^ CAMS Oxford Institute, University of Oxford, Oxford, United Kingdom; ^7^ Oxford Kidney and Transplant Unit, Oxford University Hospitals NHS Foundation Trust, Oxford, United Kingdom; ^8^ Division of Dermatology, The Ohio State University, Columbus, OH, United States; ^9^ Department of Dermatology, Dartmouth Hitchcock Medical Center, Lebanon, NH, United States; ^10^ Department of Dermatology, Yale School of Medicine, New Haven, CT, United States; ^11^ Schweiger Dermatology Group, New York, NY, United States; ^12^ Division of Dermatology, Department of Internal Medicine, Dell Medical School, University of Texas, Austin, TX, United States; ^13^ Department of Dermatology, Penn State Health, Penn State Milton S. Hershey Medical Center, Hershey, PA, United States; ^14^ Department of Dermatology, Johns Hopkins University School of Medicine, Baltimore, MD, United States; ^15^ Department of Dermatology, Roswell Park Comprehensive Cancer Center, Buffalo, NY, United States; ^16^ Department of Dermatology, The Netherlands Cancer Institute, Amsterdam, Netherlands; ^17^ Department of Dermatology, Leiden University Medical Centre, Leiden, Netherlands; ^18^ Molecular and Clinical Medicine, School of Medicine, University of Dundee, Dundee, United Kingdom; ^19^ Department of Dermatology, University of Pittsburgh, Pittsburgh, PA, United States; ^20^ Department of Dermatology, Duke University School of Medicine, Durham, NC, United States; ^21^ Department of Dermatology, University Hospitals Cleveland Medical Center, Case Western Reserve University School of Medicine, Cleveland, OH, United States

**Keywords:** skin cancer, squamous cell carcinoma, transplant assessment, melanoma, dermatology, triage, scheduling, SUNTRAC

## Abstract

Solid organ transplant recipients (SOTRs) have a high risk of developing aggressive skin cancers. However, there are no standardized triage guidelines to assist dermatology clinics with scheduling new patients pre- or post-transplant. Dermatologic care of SOTRs requires multidisciplinary coordination, extensive assessment, tailored counseling, and longitudinal care. Specialized high-risk transplant clinics are designed to address this clinical need but are a limited resource. This triage algorithm aims to provide a practical framework for tertiary care centers or community practice clinics receiving pre- or post-transplant referrals for active concerning growths or routine skin cancer screening exams. In summary, our expert panel recommends SOTRs are seen within 1–2 weeks for evaluation of an active growth and triaged according to their risk factors for the initial post-transplant screening visit (6 months–2+ years post-transplant). Transplant candidates should be seen for pre-transplant evaluation within 1 month of the referral for a skin cancer screening exam, depending on the transplant team’s timeline and dermatologist availability. Overall, dermatologists face numerous challenges in caring for transplant patients, and scheduling these patients in a timely manner according to the acuity of their needs will facilitate prevention and early diagnosis of skin cancer, thus improving transplant patient outcomes.

## Graphical Abstract

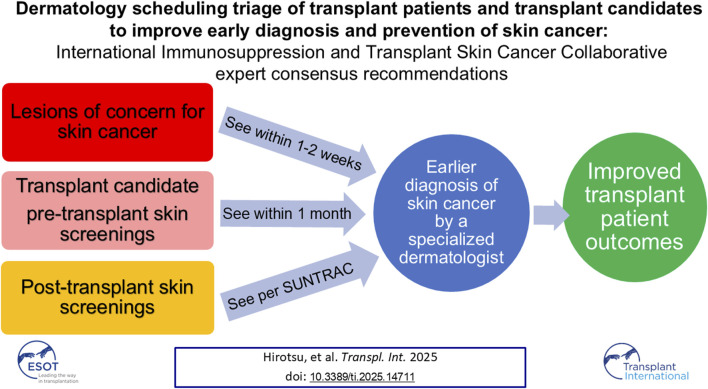

## Introduction

Solid organ transplant recipients (SOTRs) have a much greater risk of developing aggressive skin cancers due to chronic immunosuppression including a 65 to 250-fold increased risk of developing squamous cell carcinoma compared to the general population [1, 2]. Additionally, patients who are transplant candidates awaiting an organ or undergoing evaluation for an organ should be seen in a timely manner to screen for skin cancers that can affect transplant candidacy [3, 4]. The Skin and Ultraviolet Neoplasia Transplant Risk Assessment Calculator (SUNTRAC) was developed to stratify SOTRs by skin cancer risk for post-transplant screening guidelines. SUNTRAC and a published Delphi consensus for initial skin cancer screenings have together established guidelines for screenings post-transplant, but do not provide a recommended timeline for scheduling transplant patients with active concerning growths, or for those needing pre-transplant skin screening [5, 6].

Comprehensive dermatologic management of transplant patients to reduce their risk of cutaneous malignancy requires a complex level of care including multidisciplinary coordination and communication, longitudinal care from a pre-transplant risk assessment to long-term follow-up screenings, and management of topical and oral chemoprophylaxis. This level of care can be provided in the community or at tertiary care dermatology clinics in the form of high-risk transplant clinics (HRTCs). HRTCs are specifically designed to address this clinical need; however, patient access can be challenging as these specialist resources are limited. The goal of this triage algorithm is to provide a practical framework for dermatology clinic schedulers and non-specialized providers when receiving new patient referrals for transplant recipients and transplant candidates with an active concerning growth or for routine skin cancer screening.

## Methods

To frame expert recommendations, we formed an expert panel comprised of 17 physicians: 14 from the United States, two from the United Kingdom, and one from the Netherlands (Table 1). 15 experts currently practice at academic centers. Experts were defined as board-certified dermatologists or transplant physicians with at least 5 years of experience caring for SOTRs with skin cancer. A literature review was conducted to identify relevant prior research studies through a comprehensive key word search of PubMed, Embase, and MEDLINE for the following terms: triage; scheduling; transplant; immunosuppression; skin cancer; screening; guidelines. The panel determined that the low levels of evidence available would be augmented by this expert consensus study design to define best practice.

**TABLE 1:** Demographics of the expert panel.

Characteristics	Number (%)
Country	
United States	14 (82%)
Northeast	6 (35%)
Southeast	2 (12%)
Southwest	1 (6%)
Midwest	3 (18%)
West	2 (12%)
United Kingdom	2 (12%)
Netherlands	1 (6%)
Academic Center	
Yes	15 (88%)
No	2 (12%)
Specialty	
Dermatologic Surgery	9 (53%)
Medical Dermatology	7 (41%)
Transplant Nephrology	1 (6%)

To address this clinical practice gap in our transplant patient population, this agenda item was presented to and discussed by attendees at the 2024 International Immunosuppression and Transplant Skin Cancer Collaborative (ITSCC) symposium, which included ITSCC and Skin Care in Organ Transplant Patients- Europe (SCOPE) members. ITSCC and SCOPE are physician organizations that perform integrative collaborative research focused on transplant recipients and their risk of cutaneous malignancy. Attendees of the symposium included Mohs micrographic and dermatologic oncology surgeons, medical dermatologists, medical oncologists and nephrologists. Table 2 was presented to the attendees and feedback was collected and incorporated into our triage algorithm, which was distributed to the expert panel and underwent two iterations before a finalized version was approved by all authors. In the first iteration, the revised consensus recommendations in Table 2 were provided to the panelists for open-ended feedback, which was subsequently integrated by authors KEH and BTC. In the second iteration, the manuscript and all tables and figures were presented to the panelists. After obtaining an expert consensus, the recommendations were approved by the ITSCC Board of Directors.

**TABLE 2:** Recommended dermatology scheduling triage of solid organ transplant recipient and transplant candidate new patient referrals.

Urgency: Acute → Less acute	Tertiary dermatology clinic with a HRTC	Tertiary dermatology clinic without a HRTC	Community dermatology clinic
SOTR with an active growth (rapidly growing, tender or painful, or easily bleeding)	See within 1–2 weeks with HRTC, MMS or general dermatology	See within 1–2 weeks with MMS or general dermatology	See within 1–2 weeks for biopsy with MMS or general dermatology, or urgently route directly to the nearest tertiary dermatology clinic to be seen within 2 weeks Consider referral to the nearest tertiary dermatology clinic (ideally with a HRTC) for co-management
Transplant candidate pre-transplant screen	See within 1 month^a^ for screening with HRTC if available, or general dermatology	See within 1 month^a^ for screening with general dermatology, or consider referral to the nearest HRTC	See within 1 month^a^ for screening with general dermatology Consider referral to the nearest tertiary dermatology clinic (ideally with a HRTC) for co-management
SOTR with a history of skin cancer	See within 6 months-2+ years post-transplant^b^ with HRTC.	See within 6 months-2+ years post-transplant^b^ Consider long-term co-management with the nearest HRTC if available	See within 6 months-2+ years post-transplant^b^ Consider referral to the nearest tertiary dermatology clinic (ideally with a HRTC) for co-management
SOTR with no history of skin cancer	See within 1–5 years post-transplant^b^ with HRTC.	See within 1–5 years post-transplant^b^ with general dermatology	See within 1–5 years post-transplant^b^ with general dermatology

SOTR, solid organ transplant recipient; HRTC, high-risk transplant clinic; MMS, Mohs micrographic surgery/surgeon.

^a^ This recommendation can be tailored to each individual patient case, depending on the transplant team’s timeline and dermatologist availability.

^b^ Refer to SUNTRAC for the recommended timeline for the initial skin cancer screening visit [5].

## Results

### Suggested Scheduling Timeline for Solid Organ Transplant Recipients and Transplant Candidates

Real-world experience from the expert panel determined that the most critical transplant-related new patient referrals were for transplant recipients with active concerning growths. Other time-sensitive scheduling matters included pre- and post-transplant skin screening exams (Table 2). We defined three clinical settings that receive these new patient referrals: tertiary dermatology clinics with a high-risk transplant clinic (HRTC), tertiary dermatology clinics without a HRTC, and community dermatology clinics. The panel determined that both the timelines for scheduling new patients and the appropriate type of physician can vary based on the reason for referral and the clinical setting. While ideally all patients would have access to a tertiary dermatology center with a HRTC, the triage algorithm reflects recommendations but not guidelines given the limitation of this resource and potential barriers to patient access. The HRTC model, components of the pre-transplant screening visit, and a suggested approach to follow-up after the initial visit are outlined in subsequent sections of the results.

Based on the identified clinical need and resource availability challenges defined above:

1. We recommend that SOTRs with an active concerning growth (lesion that is rapidly growing, tender or painful, or easily bleeds) are seen by a dermatologist, preferably in a HRTC if available, within 1–2 weeks of receiving the referral. All referrals for concerning growths should include high-quality photographs of the growth, ideally one from close-up to demonstrate lesion morphology and one from further away to demonstrate relevant anatomic landmarks. These referrals may be triaged via teledermatology prior to an in-person visit, and lesions concerning for invasive squamous cell carcinoma or other aggressive cutaneous malignancies on teledermatology should be seen within 1–2 weeks.
2. We recommend that transplant candidates are seen within 1 month of receiving a referral for a pre-transplant skin cancer screening exam (see “*the pre-transplant screening visit”* section below). If a pre-transplant screening is not feasible or is not performed, the patient can be seen within the first year after transplant for this visit. If the patient is admitted to the hospital and expected to be hospitalized until the transplant takes place, the skin cancer screening exam can take place in the inpatient setting if possible.
3. We recommend that SOTRs referred for routine skin screenings without active concerning growths should be seen according to the SUNTRAC and/or Delphi consensus guidelines [5, 6]. All referrals to dermatology should include patient race, sex assigned at birth, history of skin cancer (yes/no), age at time of transplant, and organ transplant type, to fulfill the criteria for the SUNTRAC tool.
4. For SOTRs seen by a community dermatologist, consider referral to a HRTC for co-management of patients with a history of skin cancer, a growth that is concerning for skin cancer, or if the actinic burden is high to discuss chemoprevention and non-surgical management.

### High-Risk Transplant Center Clinical Model

HRTCs provide comprehensive dermatologic care for transplant patients in an ideal model that is highly dependent on resource availability and not feasible in every practice setting [7–9]. In general, transplant dermatologists offer screening exams, field treatments, oral chemoprevention, surgical and non-surgical treatments for skin cancer, access to multispecialty care for management of high-risk skin cancers, and coordination with the primary transplant team to manage systemic immunosuppression to mitigate skin cancer risk. Counseling for all patients includes patient-specific skin cancer risk assessment and prevention. Additionally, these clinics should have the capacity for urgent add-on visits for lesions concerning for invasive squamous cell carcinoma or other aggressive cutaneous malignancies ideally within 1–2 weeks. Although most dermatologists who provide this comprehensive transplant care are at dedicated HRTCs, community or academic dermatologists who specialize in transplant dermatology can also meet this ideal clinic model and serve as a valuable resource for transplant patients.

### The Pre-Transplant Screening Visit

The pre-transplant screening visit should include a comprehensive review of the patient’s pertinent medical history, thorough physical exam, and appropriate counseling, either within a HRTC (if available) or general dermatology clinic depending on the patient’s history of skin cancer (Table 3). If the pre-transplant screening visit has not been conducted or is not feasible with the transplant team’s timeline, the components of this visit should be included with the first post-transplant visit. Regarding the medical history review, this should include the patient’s history of melanoma and non-melanoma skin cancer, reviewing relevant previous pathology records and confirming all skin cancers were appropriately treated, any history of risk factors for skin cancer (i.e., history of extensive UV exposure including tanning bed use, actinic keratoses and prior field treatments, HPV-associated lesions, radiation therapy, relevant family history), and history of immunosuppression.

**TABLE 3:** A comprehensive pre-transplant screening visit checklist for transplant dermatologists.

Components of the visit	Patient questions
Medical History	1. Does the patient have an extensive history of UV exposure including sunburns or tanning bed use? 2. Does the patient have a history of melanoma and/or non-melanoma skin cancer? 3. When and how were all skin cancers treated? (Assess the trend of new skin cancers diagnosed per year and obtain/review relevant previous pathology records.) 4. Does the patient have a history of risk factors for skin cancer (i.e., actinic keratoses and prior field treatments, HPV-associated lesions, history of radiation therapy, relevant family history)? 5. Does the patient have a history of immunosuppression (i.e., hematologic malignancy, HIV, or long-term immunosuppression medication exposure for autoimmune or inflammatory diseases)?
Physical Exam	1. In-person head-to-toe mucocutaneous examination for lesions concerning for malignancy, pre-malignancy, or infection. Biopsy as needed 2. Overall photodamage assessment 3. Consider palpation of relevant lymph node basins based on patient history
Counseling	1. Discuss a preventative therapy plan including sun protective measures 2. Based on the patient history and physical exam, discuss potential future therapies including topical and/or oral treatment to mitigate skin cancer risk

**FIGURE 1:**
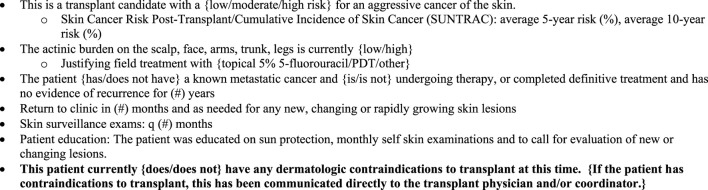
Example of a pre-transplant dermatologic assessment communication back to the referring transplant team.

For patients with a history of melanoma, Merkel cell carcinoma, and high-risk squamous cell carcinoma, a consensus opinion from ITSCC has been published for recommended waiting periods prior to transplant in 2016 [4]. More recently in 2021, the delay time for patients with a pre-transplant diagnosis of melanoma has been revised by the American Society of Transplantation [10]. The physical exam should ideally be in-person, including mucosal regions (oral and genital) and an overall photodamage assessment. Counseling can be introductory and tailored to the patient to include a preventative therapy plan and education regarding skin cancer risk and prevention. Following the pre-transplant visit screening, referring transplant teams should receive documentation from the visit including any necessary delay time, the treatment status and risk profile of any known skin cancers, actinic burden and any plans for field therapy and/or chemoprevention, and surveillance recommendations (Figure 1).

To determine transplant candidacy for each individual patient, the primary transplant team has numerous tasks to complete. Dermatologists play a critical role in the pre-transplant screen as the most qualified providers to fully assess a patient’s eligibility based on their history of skin cancer and their current skin exam. Therefore, providing a pre-transplant screening visit in a timely manner is a valuable contribution to the transplant team and establishes a patient-provider relationship that will continue post-transplant. While we recommend seeing these patients within 1 month of receiving the referral from the primary transplant team as a professional courtesy, the timeline can be tailored to each patient’s case depending on the transplant team’s timeline and dermatologist availability. Furthermore, follow-up dermatology visits may be indicated if the patient is waiting for an organ for an extended period.

### Follow-Up After High-Risk Transplant Clinic Evaluation

After a transplant patient or transplant candidate has undergone an in-person evaluation by a dermatologist at a HRTC, subsequent follow-up appointments may be performed either at the HRTC or by their local dermatologist. The frequency of follow-up visits and the recommendation for an appropriate physician is based on the high-risk transplant dermatologist’s discretion. These will be patient-specific and will depend on the patient’s skin cancer risk factors and practical considerations, including travel, patient preference, and out-of-pocket costs. For patients deemed at low risk for skin cancer or with barriers to frequent HRTC visits, follow-up visits can be scheduled alternating between HRTC and their local community dermatologist or with their local community dermatologist only and HRTC as needed.

## Discussion

Dermatologic management of transplant patients requires multidisciplinary coordination, extensive assessment and tailored counseling, and longitudinal care from pre-transplant screening visits to routine follow-up skin exams. The spectrum of care required will vary, often significantly, from patient to patient. Dedicated HRTCs are a specialized, valuable resource intended to provide optimal dermatologic management for our transplant patients and triage patients for lower acuity follow-up after initial evaluations. Access to care remains a concern for many patients. Therefore, coordination between local dermatologists and HRTCs serves an indispensable role for patients with a low risk of skin cancer and those who live far from HRTCs. Although limitations of our recommendations include the lack of diversity of global representation of the expert panel and a restricted thematic analysis, overall dermatologists face numerous challenges in caring for SOTRs, and scheduling these patients in a timely manner according to patient needs will aim to improve prevention and early diagnosis of skin cancer.

Given the increased risk of skin cancer in SOTRs, these triage recommendations aim to ensure prompt access to dermatologic services in order to reduce the skin cancer burden and progression of high-risk skin cancers in SOTRs with the ultimate goal of improving patient morbidity and mortality. Additionally, the benefits of screening and appropriate triage may include reduction of overall healthcare costs, as transplant recipients have a higher cost of skin cancer-related care relative to nonimmunosuppressed patients [11, 12]. Early assessment and intervention with the pre-transplant screening visit will serve as primary prevention to alter the trajectory of skin cancer risk in future transplant patients. Furthermore, timely appointments with the appropriate physician for SOTRs with lesions concerning for aggressive skin cancer will mitigate risks of complications such as metastatic spread (secondary prevention) through early diagnosis of potentially high-risk skin cancers. Lastly, these recommendations address post-transplant initial skin cancer screening surveillance timelines based on the evidence-based SUNTRAC guidelines. Together, these preventative aims of the scheduling triage algorithm present multiple stepwise opportunities to mitigate the impact of skin cancer in SOTRs, thus improving transplant patient outcomes.
